# Traffic-Related Trace Element Accumulation in Roadside Soils and Wild Grasses in the Qinghai-Tibet Plateau, China

**DOI:** 10.3390/ijerph110100456

**Published:** 2013-12-30

**Authors:** Guanxing Wang, Xuedong Yan, Fan Zhang, Chen Zeng, Dan Gao

**Affiliations:** 1 MOE Key Laboratory for Urban Transportation Complex Systems Theory and Technology, Beijing Jiaotong University, Beijing 100044, China; E-Mails: 12121009@bjtu.edu.cn (G.W.); 11121095@bjtu.edu.cn (D.G.); 2 Key Laboratory of Tibetan Environment Changes and Land Surface Processes, Institute of Tibetan Plateau Research, Chinese Academy of Sciences, Beijing 100101, China; E-Mails: zhangfan@itpcas.ac.cn (F.Z.); zengchen@itpcas.ac.cn (C.Z.)

**Keywords:** Qinghai-Tibet Plateau, trace element, roadside soil, roadside grass, transfer factor (TF)

## Abstract

This research examines traffic-source trace elements accumulations and distributions in roadside soils and wild grasses in the Qinghai-Tibet Plateau. A total of 100 soil samples and 100 grass samples including *Achnatherum splendens*, *Anaphalis nepalensis*, *Artemisia sphaerocephala*, *Carex moorcroftii*, *Iris lacteal*, *Kobresia myosuroides*, *Oreosolen wattii*, *Oxytropis ochrocephala* and *Stellera chamaejasme* were collected at 100 sites from different road segments. The contents of metals and metalloids, including Cu, Zn, Cd, Pb, Cr, Co, Ni and As, in the soil and grass samples were analyzed using ICP-MS. The total mean concentrations of the eight trace elements in soils are Cu (22.84 mg/kg), Zn (100.56 mg/kg), Cd (0.28 mg/kg), Pb (28.75 mg/kg), Cr (36.82 mg/kg), Co (10.24 mg/kg), Ni (32.44 mg/kg) and As (21.43 mg/kg), while in grasses are Cu (9.85 mg/kg), Zn (31.47 mg/kg), Cd (0.05 mg/kg), Pb (2.06 mg/kg), Cr (14.16 mg/kg), Co (0.55 mg/kg), Ni (4.03 mg/kg) and As (1.33 mg/kg). The metal and metalloid concentrations in the nine grass species were all below the critical values of hyperaccumulators. The mean values and Multivariate Analysis of Variance (MANOVA) results indicate that: (1) the concentrations of the trace elements in the soils are higher than those in the grasses, (2) the concentrations of Cu, Zn, Cd, Pb in the soils decrease as the roadside distance increases, (3) the concentrations of trace elements in the grasses are the highest at 10 m from the road edge, (4) the higher the traffic volume, the higher the concentrations of the trace elements in the roadside soils and grasses, and (5) when the land cover is meadow, the lower the sand content in the soil, the lower the trace element concentrations. With a trace element’s bioavailability represented by its transfer factor (TF) from the soil to the grass, the TFs of the eight trace elements are not in the same orders for different grass species.

## 1. Introduction

Soils are recognized to be major sinks for various of contaminants [1,2]. Except for organic contaminants, heavy metals such as Cu, Zn, Pb, Cd, Cr, Co, Ni and the metalloid As (collectively known as metal(loid)s or trace elements) [1] in soils have become a growing concern in recent years because of their toxicity and persistency in the environment [3]. Traffic activity is the main source of metal(loid)s emissions to roadside soils [4,5,6] and higher metal(loid) concentrations can be found in the soils and road dust along the roads with heavy traffic [3,6,7].

The metal(loid)s can be absorbed by plants, and possibly harm human health through the food chain [8]. Some metals, such as Cu, Zn and so on, are necessary for plants, and they play a very important role in plants growth. However, trace elements in soils will harm plants when their concentrations are over certain critical values. Despite this, some kinds of plant species called hyperaccumulators can grow well in soils with high concentrations of trace elements, such as in mining areas [9,10]. In recent decades, scholars have found many hyperaccumulators in wild areas [11]. Hyperaccumulators can be used to clean the metal(loid)s in polluted soil [12,13]. Therefore, research on trace element accumulation caused by road traffic and their uptake by plants are of great significance to the region’s environmental protection and human health safety.

Called as “the Roof of the World” and “the Third Pole of the World”, the Qinghai-Tibet Plateau is the highest and biggest plateau of the World, occupying an area of 2.5 million km^2^ and with an average elevation of over 4,000 m above sea level (a.s.l.) [8,14,15,16]. With the development of the Chinese economy and improved transportation conditions, its unique geographical position and special climate environment have attracted more and more tourists. The environment has been gradually affected by this activity. Because it is rarely affected by other anthropogenic activities [8], such as industrial activities, traffic is the main source of metal(loid)s in soil along the roads there. In other words, the Qinghai-Tibet Plateau is a suitable place to investigate the relationship between traffic and related environment contamination.

In this study, grass samples were collected from the same sites where the soil samples were collected. The metal(loid) concentrations in the soils and the relationship to the roadside distance has been discussed in a previous study of Yan *et al.* [8], and this research will mainly discuss: (1) the correlation of the metal(loid)s in soils and grasses, (2) the relationship between roadside distance, land cover, traffic volume and the concentrations of the trace elements in soils, as well as in grasses, (3) whether the grasses selected in this research are hypheraccumulators, and (4) the transfer factors (TF) of the trace elements from the soil to the grass.

## 2. Materials and Methods

### 2.1. Study Area

**Figure 1:**
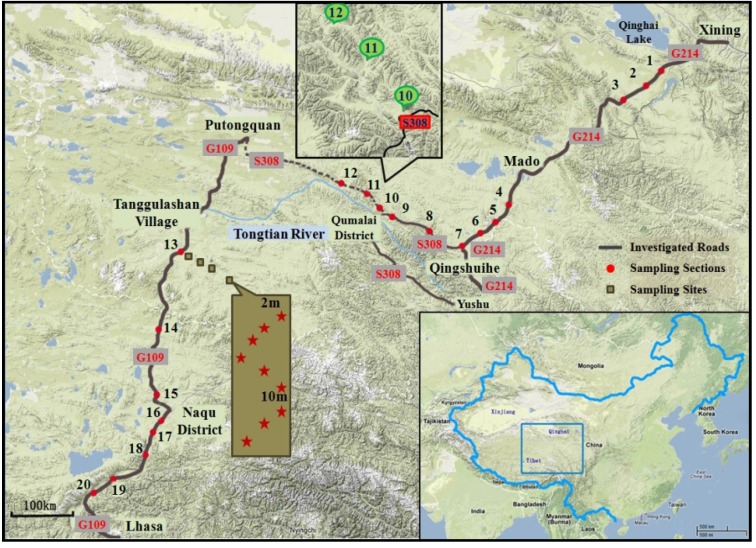
Location of the three roads and sampling sites.

One hundred samples were collected from 20 sampling sections along three roads as shown in Figure 1 (coordinates: 30°15'0.51"N–36°4'2.24"N, 90°39'13.13"E–100°33'11.95"E; Altitude: 2,771 m–5,000 m a.s.l.) on the Qinghai-Tibet Plateau from July to August 2011. The profiles of the sampling sections are shown in Table 1. The three roads are the #214 national highway (G214) from Xining to Qingshuihe, the #308 provincial highway (S308) from Qingshuihe to Putongquan and the #109 national highway (G109) from Putongquan to Lhasa. G109 is the most important transportation route into the Qinghai-Tibet Plateau. G214 is named the most beautiful national highway due to the biodiversity, geological diversity, and landscape diversity along the road. S308 is a link between G214 and G109. Some sections of S308 were damaged in the 2010 Yushu earthquake and have been repaired afterwards. During the sampling time period, traffic observation was implemented by counting the traffic volume for 1 h. Accordingly, the rank of the traffic volumes of the three roads is speculated as G214 > G109 > S208.

**Table 1:** Sampling section profiles.

Road	G109	S308		G214
No. of the road sections	1–7	8–9	10–12	13–20
Traffic Volume	Medium	Medium	Low	High
Altitude (m a.s.l.)	3,771–4,500	4,000–4,500		4,500–5,000

### 2.2. Soil Sampling and Processing

As shown in Figure 1, the distance between each of the 20 sampling sections is 20 km at least. For each section, five sampling sites were chosen at the distance of 0 m, 10 m, 30 m, 50 m and 100 m respectively from the sampling site perpendicular to the road edge. At each site, 8–10 sub-samples were taken in an “S-shape” pattern in a 10 m × 2 m plot and evenly mixed [16]. A ditch of 5 cm in depth and 10 cm in diameter was dug for each sub-sample. After digging and mixing, the soil sample was put into a zip lock bag. 

A total of 100 topsoil samples were collected. In order to analyze the correlation between trace elements and other conditions, three independent variables (DIST, LAND COVER and VOLUME) were recorded during sample collection. The sample size and description of variables related to the soil samples are shown in Table 2, in which N represents the number of samples.

**Table 2:** Sample size and description of variables related to the soil samples.

Independent Variable	N	Variable Definition	Discrete Level
DIST	20	The distance from the sampling location perpendicular to the road edge	Level 1: 0 m
	20		Level 2: 10 m
	20		Level 3: 30 m
	20		Level 4: 50 m
	20		Level 5: 100 m
LAND COVER	45	Land cover different types of land cover	Level 1: Meadow soil
	30		Level 2: High sandy meadow soil
	25		Level 3: Low sandy meadow soil
VOLUME	40	Three levels of traffic volume	Level 1: HIGH—traffic volume above 200 vehicles per hour;
	15		Level 2: LOW—traffic volume below 50 vehicles per hour;
	45		Level 3: MEDIUM—— traffic volumebetween 50 and 200 vehicles per hour.

In the laboratory, the soil samples were air dried and milled by an agate mortar to pass through a <0.15 mm nylon sieve. For metal analyzing, 0.3 ± 0.0001 g of soil sample was taken in digestion tube with 9.25 mL of acid mixtures (6 mL HNO_3_ + 3 mL HCL + 0.25 mL H_2_O_2_) added. The digestion tube was then put in an automatic microwave and heated gradually to 120 °C, holding for 5 min, then to 160 °C, holding for another 5 min, and at last to 190 °C, holding for 40 min. After complete digestion, the extract was first diluted to 50 mL with deionized water, and then filtered. One mL of the filtered dilution was then diluted 10 mL. Consequently, the concentrations of trace elements (Cu, Zn, Cd, Pb, Cr, Co, Ni and As) were determined using the Inductively Coupled Plasma-Mass Spectrometry (ICP-MS, Thermo X Series 2, Thermo Fisher Scientific Inc., Waltham, MA, USA). In every batch of 40 samples for ICP-MS analyze there were two (5%) blank reagents and two (5%) standard reference soil materials (The National Standard Substance GBW-07407 and Soil Standard Reference Sample No. GSS-7) included for quality control. The relative standard deviation was less than 10 % [16].

### 2.3. Grass Sampling and Processing

**Figure 2:**
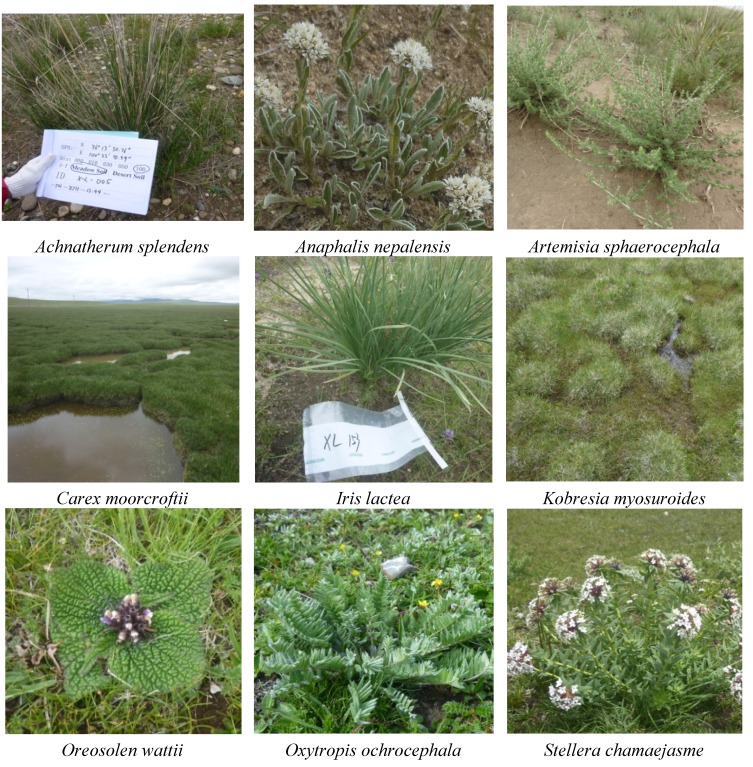
Photos of the selected grass species.

As shown in Figure 2, nine species of grasses (*Achnatherum splendens*, *Anaphalis nepalensis*, *Artemisia sphaerocephala*, *Carex moorcroftii*, *Iris lacteal*, *Kobresia myosu*roides, *Oreosolen wattii*, *Oxytropis ochrocephala* and *Stellera chamaejasme*) were selected in this study, with consideration of the following two primary factors: (1) most of these grasses are endemic plants in the Qinghai-Tibet Plateau; (2) the grasses are abundant at the sampling site. A total of 100 grass samples were collected using a wood scissor from the same sites where the soil samples were collected. The sample size and description of variables related to each grass sample are shown in Table 3.

**Table 3:** Sample size and description of variables related to each grass sample.

Name of the Grasses		*Achnatherum splendens*	*Anaphalis nepalensis*	*Artemisia sphaerocephala*	*Carex moorcroftii*	*Iris lactea*	*Kobresia myosuroides*	*Oreosolen wattii*	*Oxytropis ochrocephala*	*Stellera chamaejasme*
Family		*Gramineae*	*Compositae*	*Compositae*	*Cyperaceae*	*Iridaceae*	*Cyperaceae*	*Scrophulariaceae*	*Fabaceae*	*Thymelaeaceae*
Genus		*Achnatherum*	*Anaphalis*	*Artemisia*	*Carex*	*Iris*	*Kobresia*	*Oreosolen*	*Oxytropis*	*Stellera*
DIST	0 m	1	4	1	3	1	4	1	4	1
	10 m	1	4	1	3	1	4	1	4	1
	30 m	1	4	1	3	1	4	1	4	1
	50 m	1	4	1	3	1	4	1	4	1
	100 m	1	4	1	3	1	4	1	4	1
LAND COVER	Level 1	0	5	0	15	0	15	5	5	0
	Level 2	5	10	0	0	0	5	0	5	5
	Level 3	0	5	5	0	5	0	0	10	0
VOLUME	HIGH	0	15	0	5	5	5	5	0	5
	LOW	0	5	0	0	0	5	0	5	0
	MEDIUM	5	0	5	10	0	10	0	15	0
TOTAL		5	20	5	15	5	20	5	20	5

Grass samples were firstly washed with deionized water, dried at 100 °C for 24 h, then grounded into powder. 0.3 ± 0.0001 g of sub-samples were digested. The analysis process of trace elements in grasses is the same as that in soil samples. For quality control during the trace elements analysis, 5% blank reagent samples and 5% standard reference plant material samples (The national standard substance GBW-10011, CRM Wheat: No. GSB-2) were randomly added to the 100 grass samples. The relative standard deviation was less than 10%.

### 2.4. Data Analysis Methods

Multivariate Analysis of Variance (MANOVA), Pearson correlation analysis, principal component analysis and cluster analysis were carried out using SPSS 20.0 package (IBM, Armonk, NY, USA). The significant affect of the independent variables (DIST, LAND COVER, VOLUME, and PLANT TYPE) on the eight trace elements in soils and plants is analyzed by MANOVA. Pearson Correlation analysis was used to derive statistical correlations and co-variation between trace elements. Sources discrimination of the eight trace elements in soil was performed by principal component analysis and cluster analysis.

The Transfer Factor (TF) represents the bioavailability of one trace element from soil to grass [16,17,18] calculated using Equation (1):

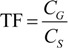
(1)
where *C_G_* is the concentration of a trace element in grass, and *C_S_* is the concentration of the trace element in the corresponding soil.

## 3. Results and Discussion

### 3.1. Trace Element Concentrations in Roadside Soil and Corresponding Grasses

Table 4 and Table 5 summarize the basic statistical descriptions of the trace element concentrations (mg/kg) in roadside soils and corresponding grasses, respectively. As shown in Table 4, the total mean values of Cu (22.84 mg/kg), Zn (100.56 mg/kg), Cd (0.28 mg/kg), Pb (28.75 mg/kg), Ni (32.44 mg/kg) and As (21.43 mg/kg) in soils were a little higher than the background values [19] except for Cr (36.82 mg/kg) and Co (10.24 mg/kg), which means that there has been some accumulation in the roadside soil. The concentrations are a little higher than the Zn (67.23 mg/kg), Cd (0.17 mg/kg), Pb (24.37 mg/kg), Ni (27.53 mg/kg) and a little lower than the Cu (23.33 mg/kg), Cr (57.77 mg/kg) and Co (11.53 mg/kg) in Zhang’s study along the Qinghai-Tibet railway [20]. Except for Pb and As, the concentrations of Cu, Cd, Cr and Ni are lower than those in Toronto, on Canada in Wiseman’s research [1]. What’s more, in Chen’s research [4] on some roadsides in Beijing (the capital of China), the concentrations of Cu (29.7 mg/kg), Pb (35.4 mg/kg) and Cr (61.9 mg/kg) were higher than those in this research, while lower for Zn (92.1 mg/kg), Cd (0.215 mg/kg), Ni (26.7 mg/kg) and As (8.1 mg/kg).

**Table 4:** Descriptive Statistics of Trace Element Concentrations in Roadside Soil (mg/kg).

			Cu-S		Zn-S		Cd-S		Pb-S		Cr-S		Co-S		Ni-S		As-S	
			Mean	S.D.	Mean	S.D.	Mean	S.D.	Mean	S.D.	Mean	S.D.	Mean	S.D.	Mean	S.D.	Mean	S.D.
DIST	0 m		26.80	5.02	114.57	32.41	0.43	0.40	41.83	31.64	37.48	11.01	10.96	2.01	37.09	24.38	21.99	4.91
	10 m		22.74	4.04	104.68	26.22	0.28	0.13	28.85	12.97	37.37	10.14	10.30	1.88	31.66	8.40	22.03	6.36
	30 m		22.48	4.14	98.86	16.72	0.26	0.12	25.76	9.21	37.59	9.14	10.29	1.73	32.11	7.20	21.89	6.36
	50 m		20.90	4.35	93.01	16.04	0.22	0.09	24.64	9.14	34.81	6.07	9.89	1.77	30.49	5.83	20.79	5.93
	100 m		21.28	4.69	91.68	14.25	0.22	0.08	22.67	7.56	36.84	7.81	9.74	1.86	30.85	5.19	20.43	5.71
LAND COVER	Level 1		21.35	4.43	93.49	12.06	0.24	0.07	23.98	9.37	37.89	11.56	9.86	1.86	32.46	9.07	19.01	6.00
	Level 2		24.47	4.85	113.05	31.57	0.36	0.33	39.48	25.38	37.49	6.09	10.38	1.10	30.98	4.71	24.42	4.52
	Level 3		23.57	4.98	98.29	22.13	0.26	0.18	24.46	12.42	34.09	5.00	10.74	2.44	34.16	21.36	22.17	5.03
VOLUME	HIGH		21.65	5.30	110.49	31.30	0.35	0.32	40.76	23.09	39.75	11.60	9.83	1.92	32.71	10.25	23.13	7.16
	MEDIUM		24.04	4.29	94.17	12.70	0.24	0.06	20.66	3.79	35.81	6.10	10.58	1.94	30.06	4.02	20.00	4.61
	LOW		22.41	4.68	93.28	12.65	0.23	0.04	21.00	3.02	32.03	3.75	10.29	1.25	38.86	26.28	21.13	3.67
Total			22.84	4.86	100.56	23.38	0.28	0.21	28.75	17.75	36.82	8.88	10.24	1.86	32.44	12.45	21.43	5.80
Background Value		Qinghai	22.2		80.3		0.14		20.9		70.1		10.1		29.6		14.0	
		Tibet	21.9		74.0		0.08		29.1		76.6		11.8		32.1		19.7	
Mean	22.1		77.2		0.11		25.0		73.4		11.0		30.9		16.9			

**Table 5:** Descriptive Statistics of Trace Element Concentrations in Grasses (mg/kg).

		Cu-G		Zn-G		Cd-G		Pb-G		Cr-G		Co-G		Ni-G		As-G	
		Mean	S.D.	Mean	S.D.	Mean	S.D.	Mean	S.D.	Mean	S.D.	Mean	S.D.	Mean	S.D.	Mean	S.D.
DIST	0 m	9.38	5.41	33.89	14.29	0.10	0.15	3.49	5.88	15.61	16.07	0.64	1.05	4.41	4.94	1.64	2.57
	10 m	10.29	5.32	32.98	15.06	0.06	0.09	2.65	4.86	15.67	19.07	0.76	1.34	4.77	5.69	1.77	2.68
	30 m	10.29	5.63	31.16	12.45	0.05	0.06	1.90	3.24	14.98	14.01	0.55	0.72	4.08	3.52	1.30	1.41
	50 m	9.24	3.76	28.10	10.48	0.04	0.04	1.25	1.93	13.46	13.01	0.45	0.69	3.46	2.82	1.04	1.23
	100 m	10.04	7.26	31.22	15.15	0.04	0.05	1.01	1.50	11.09	7.54	0.36	0.56	3.45	2.80	0.90	1.04
LAND COVER	Level 1	8.33	3.62	33.76	11.66	0.03	0.05	1.23	3.03	10.87	10.95	0.40	0.98	3.66	4.51	1.15	2.28
	Level 2	11.59	7.41	33.31	16.87	0.09	0.13	4.18	5.51	22.43	19.56	0.88	0.99	5.12	4.38	1.93	1.81
	Level 3	10.49	5.05	25.14	9.99	0.06	0.08	0.99	1.02	10.18	5.87	0.42	0.52	3.40	2.40	0.94	0.92
VOLUME	HIGH	10.86	6.89	36.69	16.89	0.09	0.11	4.01	5.53	19.75	20.51	0.85	1.26	5.23	5.84	1.96	2.74
	MEDIUM	9.16	4.46	30.07	9.68	0.03	0.07	0.63	0.91	9.55	5.37	0.31	0.45	3.18	1.77	0.83	0.74
	LOW	9.22	3.72	21.73	3.02	0.02	0.00	1.15	1.03	13.12	4.11	0.48	0.57	3.41	2.33	1.15	1.06
PLANT TYPE	Ac.S.	5.17	1.55	14.48	3.72	0.00	0.00	0.11	0.16	7.38	3.29	0.01	0.02	1.15	0.38	0.43	0.25
	A.N.	15.26	7.18	39.29	17.36	0.13	0.14	6.90	6.44	34.31	19.38	1.64	1.39	8.93	6.25	3.59	3.10
	Ar.S.	18.94	2.84	27.85	4.72	0.16	0.13	2.14	0.51	14.91	2.52	1.00	0.24	4.11	0.60	1.75	0.35
	C.M.	7.98	2.36	39.03	14.77	0.00	0.01	0.29	0.34	9.71	7.17	0.08	0.14	2.55	0.94	0.45	0.30
	I.L.	4.59	0.40	11.16	1.89	0.04	0.02	0.14	0.32	2.85	1.52	0.00	0.00	0.17	0.15	0.19	0.22
	K.M.	7.48	2.27	27.30	8.44	0.04	0.06	1.13	1.90	11.21	6.09	0.23	0.26	2.49	1.27	0.75	0.52
	O.W.	5.15	0.75	30.26	7.00	0.04	0.03	0.89	0.55	4.83	1.36	0.03	0.04	1.54	0.27	0.18	0.19
	O.O.	10.19	3.45	29.86	6.20	0.03	0.02	1.12	1.24	9.60	5.69	0.54	0.63	4.89	2.02	1.24	1.18
	S.C.	7.45	1.41	42.75	3.96	0.03	0.01	0.45	0.63	3.69	0.90	0.10	0.08	0.80	0.19	0.38	0.12
Total		9.85	5.49	31.47	13.48	0.05	0.09	2.06	3.89	14.16	14.26	0.55	0.91	4.03	4.07	1.33	1.91

As shown in Table 5, the concentrations of each trace element in soils decreased with the increase of roadside distance, as well as Zn, Cd and Pb in grasses. However the concentrations of Cu, Cr, Co, Ni and As in grasses had the highest value at 10 m to road edge. It can be inferred that the concentrations of trace elements in the soil at the sites, where the concentrations of the corresponding trace element in grasses are the maximum value, is the most befitting concentration for grasses to absorb. With consideration of the background values of the trace elements in soil [19], it can be inferred that trace elements have already been accumulated in the roadside soil. Except for Cr, Co, Ni in soil and Zn in grasses, concentrations of other trace elements were highest when the land cover is meadow with high sand content. Additionally, for both soil and grass, concentrations were the highest when the traffic volume was at the “HIGH” level except for Cu, Co and Ni in soils. But there were no significant difference in the concentrations when the traffic volume was MEDIUM and LOW.

For the different grass species, the concentrations of Pb, Cr, Co, Ni and As in *Anaphalis nepalensis* were the highest among the nine grass species, *Artemisia sphaerocephala* showed the highest concentrations of Cu and Cd, and the concentration of Zn in *Stellera chamaejasme* was the highest. However, the concentrations of Cu, Zn and Cd in *Anaphalis nepalensis* and Zn in *Carex moorcroftii* are also considerable.

The total mean values of the eight trace elements in grasses are Cu (9.85 mg/kg), Zn (31.47 mg/kg), Cd (0.05 mg/kg), Pb (2.06 mg/kg), Cr (14.16 mg/kg), Co (0.55 mg/kg), Ni (4.03 mg/kg) and As (1.33 mg/kg). Generally, the concentrations of a trace element in hyperacumulators are 100 times higher than that in ordinary plants. Currently, a plant with its above-ground-tissues containing more than 100 mg/kg Cd, 1,000 mg/kg Cu, 1,000 mg/kg Pb, 1,000 mg/kg Co, 1,000 mg/kg Ni, or 10,000 mg/kg Zn can be called as a hyperaccumulator [9]. Accordingly, none of the nine grasses in this research can be recognized as hyperaccumulators.

### 3.2. MANOVA of Trace Element Concentrations and Independent Factors

A MANOVA is used to investigate the effects of different levels of DIST, LAND COVER, VOLUME on the trace elements in soil (see in Table 6) and the influences of different levels of DIST, LAND COVER, VOLUME, PLANT TYPE on the concentrations of trace elements in grasses (see in Table 7). The hypothesis testing in the analyses is based on the 0.1 and 0.05 significance levels. The results indicate that the factors affect the concentrations of the eight trace elements in various degrees. 

For soil, DIST has significant effects on the concentrations of Cu (*p* < 0.01), Zn (*p* < 0.01), Cd (*p* < 0.01), and Pb (*p* < 0.01), LAND COVER significantly affects the concentrations of Cu (*p* < 0.01), Zn (*p* < 0.01), Pb (*p* < 0.05), and As (*p* < 0.01), and VOLUME significantly influences the concentrations of Cu (*p* < 0.01), Zn (*p* < 0.05), Pb (*p* < 0.01), Cr (*p* < 0.05), and Ni (*p* < 0.05). The sums of the *F* values in the MANOVA stand for the significance of the total effect of a factor on various trace element concentrations. For soil, significance order of the factors is VOLUME (*F* = 44.964) ＞ LAND COVER (*F* = 34.082) ＞ DIST (*F* = 25.530), illustrating that the most significant factor influencing metal concentrations in soil is VOLUME.

For grasses, PLANT TYPE has a significant influence on all the eight trace elements (*p* < 0.01), which means that the concentrations of trace elements are distinct in the different grass species. Furthermore, DIST has significant effects on Cd (*p* < 0.05) and Pb (*p* < 0.05), LAND COVER significantly affects the concentration of Cd (*p* < 0.05), Pb (*p* < 0.01), Cr (*p* < 0.01), Co (*p* < 0.01), Ni (*p* ≤ 0.05) and As (*p* < 0.01), and VOLUME significantly influences the concentrations of Zn (*p* < 0.01), Cd (*p* < 0.05), Pb (*p* < 0.01), Cr (*p* < 0.05) and Ni (*p* < 0.01). For grass, significance order of the factors is PLANT TYPE (*F* = 90.041) ＞LAND COVER (*F* = 37.894) ＞ VOLUME (*F* = 37.148) ＞ DIST (*F* = 12.13), so that the most significant factor influencing metal concentrations in grass is PLANT TYPE.

**Table 6:** MANOVA results of trace element concentrations for Soil Samples.

Source	DIST			LAND COVER			VOLUME		
	df	F	Sig.	df	F	Sig.	df	F	Sig.
Cu-S	4	6.955	0.000 **	2	10.338	0.000 **	2	8.147	0.001 **
Zn-S	4	4.396	0.003 **	2	5.100	0.008 **	2	4.496	0.014 *
Cd-S	4	4.119	0.004 **	2	2.267	0.109	2	2.309	0.105
Pb-S	4	6.958	0.000 **	2	4.720	0.011 *	2	18.649	0.000 **
Cr-S	4	0.357	0.838	2	0.969	0.383	2	4.167	0.019 *
Co-S	4	1.393	0.243	2	2.528	0.085	2	2.334	0.103
Ni-S	4	0.959	0.434	2	1.014	0.367	2	3.481	0.035 *
As-S	4	0.393	0.814	2	7.146	0.001 **	2	1.381	0.257

* Significant at *p* ≤ 0.05; ** Significant at *p* ≤ 0.01.

**Table 7:** MANOVA results of trace element concentrations for Grass Samples.

Source	DIST			LAND COVER			VOLUME			PLANT TYPE		
	df	F	Sig.	df	F	Sig.	df	F	Sig.	df	F	Sig.
Cu-G	4	0.365	0.833	2	2.925	0.059	2	2.103	0.129	8	13.962	0.000 **
Zn-G	4	0.989	0.418	2	0.532	0.590	2	11.157	0.000 **	8	7.475	0.000 **
Cd-G	4	3.206	0.017 *	2	3.723	0.028 *	2	4.611	0.013 *	8	6.836	0.000 **
Pb-G	4	3.097	0.020 *	2	5.437	0.006 **	2	5.147	0.008 **	8	9.731	0.000 **
Cr-G	4	1.039	0.392	2	11.863	0.000 **	2	3.411	0.038 *	8	17.516	0.000 **
Co-G	4	1.166	0.332	2	5.173	0.008 **	2	2.149	0.123	8	10.958	0.000 **
Ni-G	4	0.871	0.485	2	3.105	0.050 *	2	5.998	0.004 **	8	13.878	0.000 **
As-G	4	1.397	0.242	2	5.136	0.008 **	2	2.572	0.082	8	9.685	0.000 **

* Significant at *p* ≤ 0.05; ** Significant at *p* ≤ 0.01.

### 3.3. Interrelationship Analysis of Trace Elements

Table 8 and Table 9 summarize the correlation analysis of Cu, Zn, Cd, Pb, Cr, Co, Ni and As concentrations in soils and grasses, respectively. The trace elements (Cu-Zn, Cu-Cd, Cu-Pb, Cu-Cr, Cu-Co, Cu-As, Zn-Cd, Zn-Pb, Zn-Co, Zn-As, Cd-Pb, Cd-As, Pb-Cr, Pb-As, Cr-Co, Cr-Ni, Cr-As, Co-Ni, Co-As, As-Ni ) in soil show significant correlation with each other at the 0.01 or 0.05 levels. 

Trace elements in soil can be geogenic or originated from anthropogenic inputs [1,21,22]. The significant correlations indicate the homology of the metal(loid)s pollution source. Since there is no industrial pollution at the research area, traffic is the main source of the trace elements in the soil [16,17,23]. Therefore, the significant correlation proved that the trace elements in roadside soil come from the traffic contamination.

As shown in Table 7, the concentrations in grasses of every two trace elements had significant correlation with each other at the 0.01 level. This makes a clear explanation that all the eight trace elements in the nine grass species are homological. Similar to the previous study [8,17,22,23], traffic is the main source of contamination along the road, so the high concentration of trace elements in grasses are mainly influenced by the traffic through uptake from the soil by roots and from the dust by leaves [1].

**Table 8:** Correlation analysis of the metal(loid) concentrations in soil.

Pearson Correlation	Cu-S	Zn-S	Cd-S	Pb-S	Cr-S	Co-S	Ni-S	As-S
Cu-S	1.000	0.531 ^**^	0.423 ^**^	0.392 ^**^	0.294 ^**^	0.568 ^**^	0.059	0.393 ^**^
Zn-S	0.531 ^**^	1.000	0.742^ **^	0.828^ **^	0.166	0.275^ **^	0.003	0.431^ **^
Cd-S	0.423 ^ **^	0.742^ **^	1.000	0.873^ **^	0.001	0.129	-0.071	0.344^ **^
Pb-S	0.392 ^**^	0.828 ^**^	0.873 ^**^	1.000	0.202 ^*^	0.193	0.027	0.409 ^**^
Cr-S	0.294 ^**^	0.166	0.001	0.202 ^*^	1.000	0.434 ^**^	0.490 ^**^	0.288 ^**^
Co-S	0.568 ^**^	0.275 ^**^	0.129	0.193	0.434 ^**^	1.000	0.323 ^**^	0.626 ^**^
Ni-S	0.059	0.003	0.071	0.027	0.490 ^**^	0.323 ^**^	1.000	0.210 ^*^
As-S	0.393 ^**^	0.431 ^**^	0.344 ^**^	0.409 ^**^	0.288 ^**^	0.626 ^**^	0.210 ^*^	1.000

** Correlation is significant at the 0.01 level (2-tailed); * Correlation is significant at the 0.05 level (2-tailed).

**Table 9:** Correlation Analysis of the metal(loid) Concentrations in grasses.

Pearson Correlation	Cu-G	Zn-G	Cd-G	Pb-G	Cr-G	Co-G	Ni-G	As-G
Cu-G	1.000	0.528^ **^	0.656^ **^	0.627^ **^	0.622^ **^	0.590^ **^	0.618^ **^	0.540^ **^
Zn-G	0.528^ **^	1.000	0.490^ **^	0.529^ **^	0.409^ **^	0.443^ **^	0.515^ **^	0.426^ **^
Cd-G	0.656^ **^	0.490^ **^	1.000	0.777^ **^	0.643^ **^	0.566^ **^	0.565^ **^	0.543^ **^
Pb-G	0.627^ **^	0.529^ **^	0.777^ **^	1.000	0.873^ **^	0.829^ **^	0.837^ **^	0.828^ **^
Cr-G	0.622^ **^	0.409^ **^	0.643^ **^	0.873^ **^	1.000	0.850^ **^	0.841^ **^	0.817^ **^
Co-G	0.590^ **^	0.443^ **^	0.566^ **^	0.829^ **^	0.850^ **^	1.000	0.948^ **^	0.979^ **^
Ni-G	0.618^ **^	0.515^ **^	0.565^ **^	0.837^ **^	0.841^ **^	0.948^ **^	1.000	0.948^ **^
As-G	0.540^ **^	0.426^ **^	0.543^ **^	0.828^ **^	0.817^ **^	0.979^ **^	0.948^ **^	1.000

** Correlation is significant at the 0.01 level (2-tailed).

**Figure 3:**
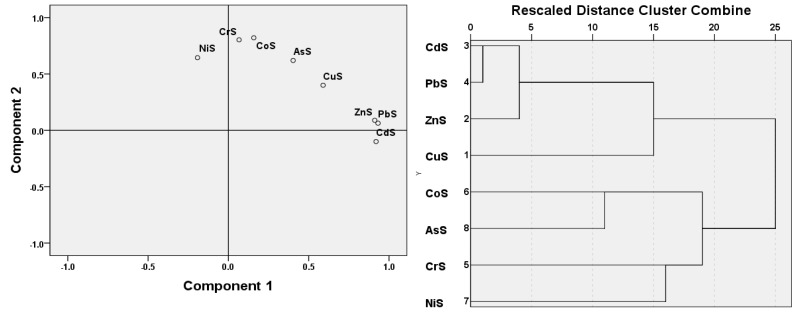
Scatter plots and cluster tree of trace elements in soil.

As in Yan *et al.*’s research [8], the eight trace elements were mostly concentrated within 30 m from the road. According to Zhang *et al.* [20], to further verify the interrelationship of the eight trace elements in soil within 30 m from the roadside and their homology, principal component analysis and cluster analysis were implemented. As a result, two components (Components 1 and 2) were extracted, where Component 1 is related to Cu, Zn, Pb, and Cd, meanwhile Component 2 is related to As, Co, Cr, and Ni. The scatter plot of factors 1 and 2 and the dendrogram in Figure 3 showed the interrelationships among the eight trace elements in soils, indicating that Co, As, Cr and Ni are geogenic, while Cu, Zn, Pb, and Cd are mainly from the traffic source, which is almost the same with studies of Shi [24], Nabulo [25] and Viard [26].

### 3.4. Transfer Factor of Trace element from Soil to Grasses

Table 10 summarizes the descriptive statistical results of Transfer Factor (TF) of the trace elements from soil to grass. According to the previous research results of TF [18,22,27,28], the value range in most cases is 0–1. In Yan’s study in Nepal [22], the TF value of Cd is above 1. In this study, the TF values are all below 1.

In total, the values of the trace element TFs are in the following order: Cu > Cr > Zn > Cd > Ni > Pb = As > Co. In Yan’s study [22] the order of TFs of the grasses in Nepal is Zn > Cu > Pb. It can be inferred that TFs varies from one grass species to another. For *Achnatherum splendens*, the order is Cr > Cu > Zn > Ni > As > Cd > Pb = Co, for *Anaphalis nepalensis*, the order is Cr > Cu > Cd > Zn > Ni > Pb > As = Co, however, for *Carex moorcroftii*, the order is Zn > Cu > Cr >Ni > As > Cd > Pb > Co.

Furthermore, *Anaphalis nepalensis* has the highest TF values for Cu, Pb, Cr, Co, Ni and As, *Artemisia sphaerocephala* has the highest TF values for Cd, while *Carex moorcroftii* and *Stellera chamaejasme* have the highest TF values for Zn.

Furthermore, the factors of DIST and LAND COVER do not show a consistent influence on TF for the trace elements. However under the high level of traffic volume, the TFs are higher than those under lower level of traffic volume.

**Table 10:** Descriptive statistical results of Transfer Factor (TF) of grass-soil trace element concentrations.

		Cu-TF		Zn-TF		Cd-TF		Pb-TF		Cr-TF		Co-TF		Ni-TF		As-TF	
		Mean	SD	Mean	SD	Mean	SD	Mean	SD	Mean	SD	Mean	SD	Mean	SD	Mean	SD
DIST	0 m	0.36	0.19	0.31	0.14	0.29	0.47	0.07	0.09	0.41	0.39	0.06	0.08	0.13	0.10	0.07	0.08
	10 m	0.46	0.24	0.32	0.13	0.19	0.26	0.07	0.10	0.40	0.44	0.07	0.11	0.14	0.14	0.08	0.10
	30 m	0.46	0.25	0.32	0.12	0.22	0.31	0.07	0.10	0.40	0.39	0.05	0.07	0.12	0.10	0.06	0.06
	50 m	0.45	0.19	0.31	0.13	0.19	0.24	0.05	0.07	0.39	0.40	0.04	0.08	0.11	0.11	0.05	0.06
	100 m	0.49	0.38	0.35	0.17	0.18	0.25	0.04	0.06	0.30	0.20	0.03	0.05	0.11	0.08	0.04	0.05
LAND COVER	Level 1	0.41	0.19	0.36	0.12	0.12	0.19	0.04	0.07	0.26	0.17	0.03	0.07	0.10	0.07	0.05	0.07
	Level 2	0.50	0.38	0.31	0.16	0.31	0.39	0.10	0.12	0.62	0.55	0.09	0.10	0.17	0.15	0.09	0.08
	Level 3	0.44	0.17	0.26	0.11	0.26	0.34	0.05	0.05	0.30	0.18	0.04	0.05	0.10	0.07	0.05	0.04
VOLUME	HIGH	0.52	0.33	0.35	0.17	0.32	0.35	0.09	0.12	0.49	0.53	0.08	0.11	0.15	0.15	0.08	0.10
	LOW	0.43	0.18	0.24	0.03	0.09	0.03	0.05	0.05	0.42	0.16	0.05	0.05	0.10	0.07	0.05	0.05
	MEDIUM	0.39	0.18	0.32	0.11	0.16	0.31	0.03	0.04	0.27	0.14	0.03	0.04	0.10	0.05	0.04	0.04
PLANT TYPE	*Achnatherum splendens*	0.19	0.07	0.15	0.06	0.01	0.01	0.00	0.01	0.22	0.12	0.00	0.00	0.04	0.01	0.02	0.01
	*Anaphalis nepalensis*	0.74	0.35	0.39	0.14	0.53	0.39	0.17	0.12	0.85	0.54	0.15	0.11	0.24	0.16	0.15	0.10
	*Artemisia sphaerocephala*	0.66	0.11	0.34	0.06	0.70	0.58	0.11	0.03	0.39	0.06	0.09	0.02	0.14	0.02	0.10	0.02
	*Carex moorcroftii*	0.37	0.14	0.41	0.13	0.02	0.04	0.01	0.01	0.26	0.19	0.01	0.01	0.09	0.03	0.03	0.02
	*Iris lactea*	0.22	0.05	0.10	0.01	0.14	0.10	0.00	0.00	0.10	0.06	0.00	0.00	0.01	0.01	0.01	0.01
	*Kobresia myosuroides*	0.33	0.11	0.27	0.12	0.12	0.22	0.02	0.03	0.32	0.16	0.02	0.02	0.08	0.04	0.04	0.03
	*Oreosolen wattii*	0.33	0.08	0.39	0.17	0.12	0.03	0.03	0.01	0.15	0.04	0.00	0.00	0.05	0.01	0.01	0.01
	*Oxytropis ochrocephala*	0.45	0.19	0.31	0.06	0.13	0.11	0.05	0.06	0.29	0.18	0.05	0.06	0.15	0.06	0.06	0.05
	*Stellera chamaejasme*	0.31	0.04	0.41	0.04	0.13	0.03	0.01	0.01	0.09	0.03	0.01	0.01	0.02	0.00	0.01	0.01
Total		0.44	0.26	0.32	0.14	0.21	0.31	0.06	0.09	0.38	0.37	0.05	0.08	0.12	0.11	0.06	0.07

## 4. Conclusions

Trace elements (Cu, Zn, Cd, Pb, Cr, Co, Ni, As) in soils and plants along the G109, S308 and G214 in the Qinghai-Tibet Plateau were investigated in this research. It was found that soil concentrations of Cu, Zn, Pb and Cd were mostly affected by the road traffic volume along the research road segments. As the research of Legret *et al.* [29], in roadside soil, Pb mostly comes from wear of the brake linings and the off gas of the vehicles, Cu almostly comes from the brake linings, and the wear and tear of the brake linings and the tires contribute evenly to Cd and Zn levels. The concentration of Pb in this research is lower than that along the researched roadside in Beijng [6] proving that the traffic volume in Qinghai-Tibet was lower than in Beijing. To the contrary, the higher concentrations of Zn and Cd in this research indicated that the condition of the road surface in Qinghai-Tibet was worse than that in Beijing, so that the tires were more worn. Considering the frequent congestion in Beijing, its higher concentration of Cu than that in Qinghai-Tibet is reasonable.

Transfer factors (TF) of the eight trace elements that represent their bioavailability vary with different grass species. Among the nine grass species, *Anaphalis nepalensis* (Pb, Cr, Co, Ni, As, Cu, Zn and Cd), *Artemisia sphaerocephala* (Cu and Cd), *Stellera chamaejasme* (Zn) and *Carex moorcroftii* (Zn) were better transfer plants, but haven’t reached the level of hyperaccumulators.

The results of this study are preliminary, but the relationship between Cu, Zn, Pb, Cd and traffic volume were verified, and the relatively high ability of *Anaphalis nepalensis* to transfer trace elements from soils were identified. They can serve as the bases for further study on long-term monitoring of trace elements accumulation in roadside soil and grasses, and on the relationship between traffic and roadside ecology, under the sensitive and vulnerable environment of the Qinghai-Tibet plateau.
